# Is late-life dependency increasing or not? A comparison of the Cognitive Function and Ageing Studies (CFAS)

**DOI:** 10.1016/S0140-6736(17)31575-1

**Published:** 2017-10-07

**Authors:** Andrew Kingston, Pia Wohland, Raphael Wittenberg, Louise Robinson, Carol Brayne, Fiona E Matthews, Carol Jagger, Cognitive Function and Ageing Studies collaboration, E Green, L Gao, R Barnes, A Arthur, C Baldwin, L E Barnes, C Brayne, A Comas-Herrera, T Dening, G Forster, S Harrison, P G Ince, C Jagger, F E Matthews, I G McKeith, B Parry, J Pickett, L Robinson, B C M Stephan, S Wharton, R Wittenberg, B Woods, R Weller

**Affiliations:** a Institute of Health and Society, Faculty of Medicine, Newcastle University, Newcastle, UK; b Newcastle University Institute for Ageing, Faculty of Medicine, Newcastle University, Newcastle, UK; c Hull York Medical School, University of Hull, Hull, UK; d Personal Social Services Research Unit, London School of Economics and Political Science, London, UK; e Department of Public Health and Primary Care, Cambridge Institute of Public Health, University of Cambridge, Cambridge, UK; f MRC Biostatistics Unit, Cambridge Institute of Public Health, University of Cambridge, Cambridge, UK

## Abstract

**Background:**

Little is known about how the proportions of dependency states have changed between generational cohorts of older people. We aimed to estimate years lived in different dependency states at age 65 years in 1991 and 2011, and new projections of future demand for care.

**Methods:**

In this population-based study, we compared two Cognitive Function and Ageing Studies (CFAS I and CFAS II) of older people (aged ≥65 years) who were permanently registered with a general practice in three defined geographical areas (Cambridgeshire, Newcastle, and Nottingham; UK). These studies were done two decades apart (1991 and 2011). General practices provided lists of individuals to be contacted and were asked to exclude those who had died or might die over the next month. Baseline interviews were done in the community and care homes. Participants were stratified by age, and interviews occurred only after written informed consent was obtained. Information collected included basic sociodemographics, cognitive status, urinary incontinence, and self-reported ability to do activities of daily living. CFAS I was assigned as the 1991 cohort and CFAS II as the 2011 cohort, and both studies provided prevalence estimates of dependency in four states: high dependency (24-h care), medium dependency (daily care), low dependency (less than daily), and independent. Years in each dependency state were calculated by Sullivan's method. To project future demands for social care, the proportions in each dependency state (by age group and sex) were applied to the 2014 England population projections.

**Findings:**

Between 1991 and 2011, there were significant increases in years lived from age 65 years with low dependency (1·7 years [95% CI 1·0–2·4] for men and 2·4 years [1·8–3·1] for women) and increases with high dependency (0·9 years [0·2–1·7] for men and 1·3 years [0·5–2·1] for women). The majority of men's extra years of life were spent independent (36·3%) or with low dependency (36·3%) whereas for women the majority were spent with low dependency (58·0%), and only 4·8% were independent. There were substantial reductions in the proportions with medium and high dependency who lived in care homes, although, if these dependency and care home proportions remain constant in the future, further population ageing will require an extra 71 215 care home places by 2025.

**Interpretation:**

On average older men now spend 2·4 years and women 3·0 years with substantial care needs, and most will live in the community. These findings have considerable implications for families of older people who provide the majority of unpaid care, but the findings also provide valuable new information for governments and care providers planning the resources and funding required for the care of their future ageing populations.

**Funding:**

Medical Research Council (G9901400) and (G06010220), with support from the National Institute for Health Research Comprehensive Local research networks in West Anglia and Trent, UK, and Neurodegenerative Disease Research Network in Newcastle, UK.

## Introduction

A common concern with increasing lifespan is that higher levels of disease will translate into a decline in capability and independence, with increased demands for health and social care services.^1^, ^2^ Increased survival to very old ages does not always result in more disability and dependency at a population level because of selective mortality of the most dependent;^3^ however, whether this observation will continue with further advances in medical technology remains unknown. Furthermore, a key challenge for ageing societies worldwide will be the projected decline in traditional sources of formal and unpaid care from families to support people living at home and in long-term residential care,^4^ and cuts to social care budgets that influence hospital discharge.^5^

The fastest growing section of the population is those aged 85 years and older,^6^ of whom up to 20% live independently and free from disability despite multimorbidity, but most will require care at some point.^7^, ^8^ In part, this requirement for care is due to the exponential increase in dementia prevalence with age, although this cause might be alleviated as reductions in dementia prevalence have been seen in the past decades in the UK and USA,^9^, ^10^ along with a substantial reduction in the years spent with cognitive impairment at least in women.^11^ By contrast, trends in disability for both countries have been less positive with a substantial increase in years with mild disability.^11^, ^12^

**Research in context**

**Evidence before this study**

We updated a previous review of trends in health expectancies, searching MEDLINE, Embase, and Web of Science from Jan 1, 2009, to Dec 31, 2016, with the search terms “healthy life years”, “free life expectancy”, “active life expectancy”, “healthy life expectancy”, “health expectancy”, “years of healthy life”, and “years with care needs”. We then excluded studies with only a single timepoint. Most studies focused on self-rated health or disability rather than dependency. Only two previous studies had estimated trends in years with care needs, both based on administrative data.

**Added value of this study**

Our measure of dependency, interval need, provides a greater transparency in the amount of care required than a simple count of activity limitations, and incorporates geriatric conditions such as incontinence and dementia, which are major predictors of long-term care use. We have shown that from age 65 years, older people can expect to spend on average between 4 years and 7·8 years with low dependency (care less than daily), 1·1 years, with medium dependency (care at set times daily), and between 1·3 years and 1·9 years with high dependency (24-h care). Current older people spend between 1·7 years and 2·4 years more with low dependency and between 0·9 years and 1·3 years more with high dependency than the generation 20 years ago.

**Implications of all the available evidence**

Our research suggests that the current social care crisis is due not only to the increasing numbers of the very old, with their higher morbidity and greater health and social care use, but that current older people are spending more of their remaining life with low and high care needs. Low care needs have implications for family and friends who supply unpaid care because this low dependency is unlikely to meet eligibility criteria for publicly funded care. High care needs have considerable implications for future provision of community services and the state provision of funding for care.

Disability profiles have been used for planning UK health and social care services but have assumed constant disability prevalence over time.^13^ Nevertheless, how disability relates to care needs is not obvious when disability is assessed by the number of activities of daily living requiring help or even when the hierarchical nature of activities^14^, ^15^ defines disability severity. A more informative measure, the interval, reflects potential need for personal and household care, surveillance, and medical-related tasks such as taking medications, with severity based on the time interval between necessary periods of help.^16^ Interval need was originally derived to measure the needs of older people for residential and community care services, and there have been a small number of surveys only, all UK based, that have estimated dependency on this basis.^8^, ^17^

Many European countries are struggling to adequately fund their social care, due in part to the economic crisis; for example, Germany has increased the contribution rate to its long-term social insurance system.^18^ Projections to 2021 of the service costs to maintain the current level of social care in England suggest an additional £940 million will be required, assuming a constant health profile of the older population.^19^ To date, there have been no comparisons that could inform the extent to which the current care crisis is due to greater levels of dependency than in previous generations, or simply more older people. Our study provides the first generational cohort comparison of the dependency profiles of people aged 65 years and older based on interval need. It uses baseline data from the two Cognitive Function and Ageing Studies (CFAS I and CFAS II) to establish whether dependency prevalence and years spent in different dependency states have changed in the past 20 years, and to model the effect of these findings on future demands for formal social care for older people in England.

## Methods

### Study design and participants

Full details of the study design, methods, and response rates have been published,^10^ and are repeated briefly here (a full description of the study design can be found in the appendix). In CFAS I, interviews were done between Aug 23, 1989, and June 13, 1994, in six geographical areas of the UK (Cambridgeshire, Gwynedd, Liverpool, Newcastle, Nottingham, and Oxford); and in CFAS II, they were done between Nov 13, 2008, and Nov 24, 2011, in three (Cambridgeshire, Newcastle, and Nottingham) of the original six geographical areas of the Medical Research Council's CFAS but with the same study design and methods. Interviews occurred only after written informed consent was obtained. Ethics approval for CFAS I and CFAS II were obtained through the multicentre ethics committee and from relevant local research ethics committees.

Participants from CFAS I were assigned as the 1991 cohort, and those from CFAS II were assigned as the 2011 cohort. The target population for each cohort was people aged 65 years or older who were permanently registered with a participating general practice in the areas (including those in institutional care), each area providing 2500 individuals aged 65 years and older with stratification by age group (65–74 years *vs* ≥75 years; 1250 per stratum per area). General practices provided lists of individuals to be contacted and were asked to exclude those who had died or might die over the next month. Oversampling was used to allow for losses (death, incorrect registration, ineligibility, general practitioner refusals, and participant or gatekeeper refusal).

### Data collection

Participants were interviewed in their normal place of residence—ie, their own home or care home. If cognitive impairment was judged to limit participants' ability to provide reliable answers, proxy informants (generally spouses, offspring, or occasionally paid-for carers) were used. Information collected in CFAS I and CFAS II included basic sociodemographics (age, sex, marital status, living arrangements, and education); cognitive status, which was assessed by the mini-mental state examination;^20^ urinary incontinence; and self-reported ability to do activities of daily living.^21^

### Assessment of dependency

Dependency was estimated with Isaacs and Neville's^16^ interval measure, which classifies participants on the basis of the lapsed time between periods when participants might require help. Four categories were used: independent (participants for whom supervision or help for any activity was not essential), low dependency (required help less often than daily), medium dependency (required help at regular intervals each day), and high dependency (required 24-h care, because help might be needed at any time, or constant supervision needed). Relevant items to define dependency level are shown in the panel. If participants were missing items but required help on higher dependency items, they were considered classifiable for dependency. We used the same allocation of activities to dependency categories as a previous study^8^ that had also validated the dependency measure against self-reported frequency of help received with activities.

Panel **Classification of dependency**

**High dependency (24-h care)**

At least one of the following: unable to get to or use the toilet (self-report), bed bound or chair bound (interviewer observed), needs help feeding (self-report or proxy rated), be often incontinent and need help dressing (self-report or proxy rated), or have severe cognitive impairment (mini-mental state examination score <10).

**Medium dependency (care at regular times each day)**

Either needs help preparing a meal (self-report) or putting on shoes and socks (self-report).

**Low dependency (care less than daily)**

At least one of the following: needs help to wash all over or bath (self-report), cut toenails (self-report), shop (self-report), or do light or heavy housework (self-report), or have considerable difficulty with household tasks, for example making a cup of tea (informant report).

**Independent**

Not classified as high, medium, or low dependency.

### Statistical analysis

We calculated age and sex-specific dependency prevalence for CFAS I and CFAS II using inverse probability weighting to account for non-response differences between the studies and study design selection.^10^ Because of small numbers with medium or high dependency, to explore time differences in dependency prevalence we combined low, medium, and high dependency and fitted logistic regression models with time (0=1991, 1=2011), age (5-year age band), sex, and region; subsequently, we further adjusted for education (0–9 years, 10–11 years, or ≥12 years) and living arrangements (lives alone, with spouse, with others in community, or in care home). Education categories were chosen to reflect statutory schooling ages 5–14 years (9 years of education) before 1947 (applicable to all the 1991 cohort), and ages 5–15 years (10 years of education) after 1947 (applicable to those approximately aged 65–80 years in the 2011 cohort). We examined whether time differences were accounted for by changes in the prevalence of diagnosed chronic disabling diseases that were self-reported: diabetes, hypertension, arthritis, chronic airway obstruction (asthma or chronic bronchitis, or both), stroke, or coronary heart disease (angina or heart attack, or both); and self-reported or interviewer observed geriatric conditions (hearing or eyesight problems).

We calculated the years lived in each of the four levels of dependency for the combined three regions common to CFAS I and CFAS II by sex for each timepoint (1991 and 2011), using Sullivan's^22^ method, which applies the prevalence of each level of dependency to a standard life table for the same period and age structure. We also calculated abridged life tables from population mid-year estimates and vital statistics death data provided by the Office of National Statistics (ONS) at the district level for the three regions and the two timepoints. Incorporating a more precise value for a_x_ (the fraction of interval lived by those dying in the interval), we calculated from national mortality data for both years (by sex) rather than assuming deaths were uniformly distributed over the interval (a_x_=0·5). Life tables were closed at age 90 years (probability of death for age group ≥90 years was 1·0). We used the standard ONS methodology^23^ to calculate 95% CIs for the health expectancies.

To project future demand for care to 2035, the proportions in each dependency state were applied to the 2014 England population projections of those aged 65 years or older, by age group (65–74 years, 75–84 years, and ≥85 years) and sex.

Health expectancies were calculated in R (version 3.0.3) and all other statistical analyses in SAS (version 9.4).

### Role of the funding source

The funders are represented on the CFAS Management Committee and the Biological Resource Advisory Committee but they had no role in study design, data collection, data analysis, data interpretation, or writing of the report. The corresponding author had full access to all data in the study and had final responsibility for the decision to submit for publication.

## Results

In the three centres combined, 7635 participants took part in CFAS I and 7796 in CFAS II with similar age distributions in both cohorts (because of age stratification). However, CFAS II, compared with CFAS I, had a higher proportion of men, and lower proportions of those living alone or in care homes or with the least amount of education (0–9 years; table 1).

**Table 1:** Sociodemographics of CFAS I and CFAS II

		**CFAS I**	**CFAS II**
N		7635 (100%)	7796 (100%)
Centre			
	Cambridgeshire	2601 (34·1%)	2558 (32·8%)
	Newcastle	2522 (33·0%)	2616 (33·6%)
	Nottingham	2512 (32·9%)	2622 (33·6%)
Gender			
	Men	3045 (39·9%)	3550 (45·5%)
	Women	4590 (60·1%)	4246 (54·5%)
Age (years)			
	65–69	1981 (25·9%)	1939 (24·9%)
	70–74	1776 (23·3%)	1873 (24·0%)
	75–79	1725 (22·6%)	1624 (20·8%)
	80–84	1308 (17·1%)	1290 (16·6%)
	≥85	845 (11·1%)	1070 (13·7%)
Living arrangement*			
	Lives alone	2903 (38·3%)	2772 (36·0%)
	With spouse	3589 (47·3%)	4205 (54·5%)
	With others in community	749 (9·9%)	535 (6·9%)
	In care home	346 (4·6%)	197 (2·6%)
Marital status*			
	Married or cohabiting	3791 (50·2%)	4363 (57·1%)
	Single	607 (8·0%)	427 (5·6%)
	Widowed	2895 (38·3%)	2280 (29·8%)
	Divorced or separated	267 (3·5%)	573 (7·5%)
Education (years)*			
	0–9	5529 (74·1%)	2052 (26·7%)
	10–11	1238 (16·6%)	3923 (51·1%)
	≥12	692 (9·3%)	1704 (22·2%)

Data are n (%) and unweighted. CFAS=Cognitive Function and Ageing Studies.

* Numbers of participants do not add up to N for CFAS I and CFAS II because of missing values in these variables. These percentages are proportions of the non-missing values.

**Figure 1:**
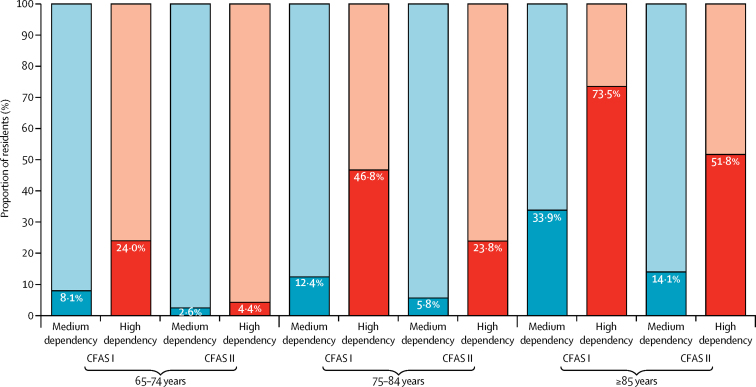
Proportions of medium dependency (care daily) and high dependency (24-h care) residents in care homes or the community for CFAS I and CFAS II Data are stratified by age and weighted. Dark blue and dark red bars are participants in care homes. Light blue and light red bars are participants in the community. CFAS=Cognitive Function and Ageing Studies.

Dependency profiles changed between 1991 and 2011, with a greater proportion of participants with low dependency (requiring care less than daily; 28·7% in 1991 *vs* 32·4% in 2011) and high dependency (24-h care; 3·9% *vs* 5·9%; appendix). Nevertheless, the proportion of older people with substantial care needs (medium or high dependency) who were living in care homes reduced considerably for all age groups (figure 1). By 2011, 51·8% of those aged 85 years or older who had high dependency were in a care home, compared with 73·5% in 1991. After adjustment for age, sex, and region, dependency prevalence was significantly higher in 2011 than in 1991 (odds ratio [OR] 1·22, 95% CI 1·15–1·30). This increase remained significant after further adjustment for cohort differences in living arrangements and levels of education (1·45, 1·34–1·56), and after adjustment for chronic disabling diseases and hearing and eyesight problems (1·21, 1·12–1·31).

**Figure 2:**
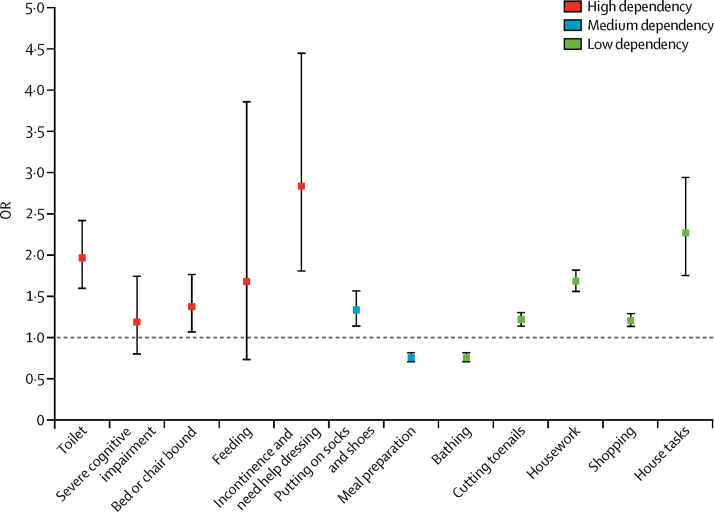
OR in 2011 compared with 1991 for individual dependency items Data are adjusted for age, sex, education, and region; and weighted. Bars are 95% CIs. Individual dependency items are of the interval need measure. OR=odds ratio.

Individual items comprising interval need were examined to ascertain which item had driven dependency changes (figure 2). Increases in high dependency over time were due to the significantly greater prevalence of help with toileting, being bed or chair bound, and being incontinent and requiring help dressing, whereas increases in low dependency over time were due to significantly higher prevalence of help required with cutting toenails, housework, shopping, and house tasks. Help with meal preparation and bathing were the only items with lower prevalence in 2011 than in 1991 (figure 2).

In 2011, average life expectancy for men aged 65 years was 17·6 years of which 11·2 years (63·5%) was spent independent, 4 years (22·9%) was with low dependency, 1·1 year (6·2%) was with medium dependency, and 1·3 years (7·4%) was with high dependency (table 2). Women aged 65 years lived longer with an average life expectancy of 20·6 years but spent fewer absolute years (and as a proportion of life expectancy) independent (9·7 years [47·3%]) and more years with low dependency (7·8 years [37·8%]). Men and women spent similar years with medium and high dependency, both in absolute years and as a proportion of life expectancy (table 2).

**Table 2:** Life expectancy and years spent in different states of dependency at age 65 years in 1991 and 2011, and differences between 1991 and 2011

		**1991**	**2011**	**Difference**
**Men**				
Life expectancy		12·9	17·6	4·7
Independent		9·5 (9·3 to 9·7)	11·2 (10·8–11·5)	1·7 (1·2 to 2·1)
Low dependency		2·3 (1·9 to 2·7)	4·0 (3·5–4·5)	1·7 (1·0 to 2·4)
Medium dependency		0·7 (0·3 to 1·2)	1·1 (0·5–1·7)	0·3 (−0·4 to 1·1)
High dependency		0·4 (−0·1 to 0·8)	1·3 (0·7–1·9)	0·9 (0·2 to 1·7)
Proportion of life expectancy spent				
	Independent	73·6% (71·8 to 75·4)	63·5% (61·4–65·6)	−10·1% (−12·9 to −7·3)
	Low dependency	17·8% (14·5 to 22·2)	22·9% (19·9–25·8)	5·1% (0·6 to 9·5)
	Medium dependency	5·8% (2·2 to 9·3)	6·2% (2·9–9·5)	0·4% (−4·4 to 5·2)
	High dependency	2·9% (−0·7 to 6·5)	7·4% (4·2–10·7)	4·5% (−0·4 to 9·3)
**Women**				
Life expectancy		16·5	20·6	4·1
Independent		9·5 (9·2 to 9·8)	9·7 (9·3–10·2)	0·2 (−0·4 to 0·7)
Low dependency		5·3 (4·9 to 5·7)	7·8 (7·3–8·3)	2·4 (1·8 to 3·1)
Medium dependency		1·0 (0·5 to 1·5)	1·1 (0·5–1·8)	0·2 (−0·6 to 1·0)
High dependency		0·6 (0·1 to 1·1)	1·9 (1·3–2·6)	1·3 (0·5 to 2·1)
Proportion of life expectancy spent				
	Independent	58·0% (56·2 to 59·9)	47·3% (45·0–49·5)	−10·7% (−13·6 to −7·8)
	Low dependency	32·4% (29·9 to 34·9)	37·8% (35·3–40·2)	5·4% (1·9 to 8·9)
	Medium dependency	5·9% (3·0 to 8·8)	5·6% (2·6–8·6)	−0·4% (−4·5 to 3·8)
	High dependency	3·7% (0·8 to 6·7)	9·3% (6·3–12·4)	5·6% (1·4 to 9·8)

Data are years (95% CI), unless specified.

Between 1991 and 2011, years spent independent after age 65 years increased significantly for men (1·7 years, 95% CI 1·2–2·1) but not for women (0·2 years, −0·4 to 0·7), and both were significantly less than the increases in life expectancy (4·7 years for men *vs* 4·1 years for women) showing an expansion of dependency. Men and women in the 2011 cohort lived more years with low dependency (1·7 years [95% CI 1·0–2·4] for men, and 2·4 years [1·8–3·1] for women) and high dependency (0·9 years [0·2–1·7] for men and 1·3 years [0·5–2·1] for women), and significantly greater proportions of life spent in these states (table 2). For men, most of their gain in life expectancy at age 65 years was spent independent (36·3%) or with low dependency (36·3%). By contrast, women had most of their gain in life expectancy with low dependency (58·0%) with only 4·8% independent.

**Figure 3:**
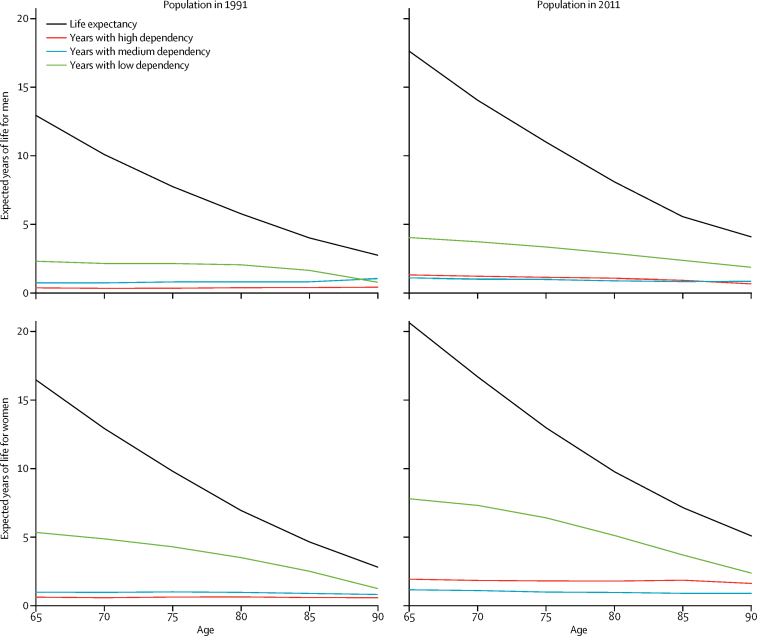
Life expectancy and years spent in different states of dependency Data are of men and women in 1991 and 2011 from the age of 65 years to older than 90 years.

By age 85 years, there were no significant differences over time in dependency states for men; however, for women, the significant increases in low and high dependency remained (appendix). Years spent with medium and high dependency are relatively constant with age (figure 3), being 1·1 years with medium dependency for both sexes in the 2011 cohort, and 1·3 years for men and 1·9 years for women with high dependency in the 2011 cohort.

If the proportion of people aged 65 years and older in each dependency state remains the same as CFAS II, then from 2015, numbers with medium dependency will increase by 190 000 by 2025 and a further 272 000 by 2035, and with similar increases in numbers with high dependency of 163 000 by 2025 and a further 237 000 by 2035 (table 3). Assuming that the proportion of those with medium and high dependency who are in care homes remains constant, these increases will require a further 71 215 care home places by 2025 and 189 043 by 2035 (table 3).

**Table 3:** Projected numbers of people aged 65 years or older and of care home places needed by 2025 and 2035

	**2015**	**2025**	**2035**	**Total increase from 2015 to 2025 (% increase)**	**Total increase from 2015 to 2035 (% increase)**
**Projected numbers of older people (thousands)**					
Independent	6705	7875	9451	1170 (17·5%)	2747 (41%)
Low dependency	3562	4447	5576	885 (24·8%)	2014 (56·5%)
Medium dependency	693	883	1155	190 (27·4%)	462 (66·6%)
High dependency	650	813	1050	163 (25·1%)	400 (61·5%)
**Projected number of care home places needed**					
Medium dependency	52 335	69 772	99 068	17 438 (33·3%)	46 734 (89·3%)
High dependency	168 376	222 154	310 686	53 777 (31·9%)	142 310 (84·5%)
Total	220 711	291 926	409 754	71 215 (32·3%)	189 043 (85·7%)

## Discussion

The past 20 years have seen continued gains in life expectancy at older ages, but not all have been healthy years, with about 2 years more spent with low dependency (requiring help less often than daily), but, more importantly, about 1 year more with high dependency (24-h care). Moreover, fewer older people with substantial care needs (medium or high dependency) now reside in care homes, although if current numbers stay constant, an extra 71 215 care home places will be needed by 2025. These findings have considerable implications for older people and their families, with the increasing diversity of family structures, and for countries struggling to fund care for their ageing populations.

Between 1991 and 2011, we found significant increases in the likelihood of requiring help for three (60%) of the five items defining high dependency, one (50%) of the two items defining medium dependency, and four (80%) of the five items defining low dependency. It is therefore unlikely that our increases in low and high dependency are driven by changes in one or two items. Despite interval need having a strong correlation with frequency of help required,^8^ the allocation of bathing or washing all over into low dependency rather than medium dependency could be debated. However, analysis of changes in the individual items suggest that moving bathing into moderate dependency would result in even greater increases in low dependency, and little change or a slight decrease in medium dependency. Nevertheless, the levels of dependency reflected in interval need provide a much more transparent indication of intensity of care required than simply summing the number of activities with which an older person requires help.

Time trends in health expectancies have generally focused on self-rated health and disability, often without any regard to severity of disability,^11^, ^24^ although there have been studies at a single timepoint reporting health expectancy based on interval need.^8^, ^17^ The USA^12^ and Japan^25^ saw increases in years spent with milder disability and stability in years with severe disability over a similar period, in keeping with our earlier findings^11^ that used a more restricted set of activity items. In our present study, years with care needs at age 65 years increased by 1·5 years per decade for men and 1·95 years per decade for women, which are substantially more than those in Germany between 2001 and 2009 (approximately 0·28 years per decade for men and 0·47 years per decade for women)^26^ and in Japan between 2005 and 2009 (0·38 years per decade for men and 0·90 years per decade for women).^27^ However, the German and Japanese studies assessed receipt of care from administrative data, which might reflect demand and could miss unmet need.

If the increase in years with low dependency continues, there will be considerable implications for family and friends who provide the majority of care at this level. Many older people feel the responsibility for care should be with the family rather than with the state, although increasing numbers of divorces, more geographically disparate families, extended working life, and increases in female labour market participation will make such provision more difficult in the future, not only in high-income countries but worldwide.^28^ Moreover, in England, local authorities have tightened their eligibility criteria for publicly funded care and therefore people with low dependency are unlikely to qualify for it; those without close family members to provide unpaid care and sufficient income to purchase care might be left with unmet needs, exacerbating inequalities.^29^ Future longitudinal analysis of CFAS will examine the extent to which the increases in low dependency have widened inequalities as a result of earlier dependency onset in the socially disadvantaged.

The past two decades have seen reductions in the overall numbers in care homes but higher prevalence of functional and cognitive impairments among residents.^30^ Our findings showed that more years were spent with high dependency, and that older people with substantial dependencies are now more likely to live supported in the community. If dependency prevalence remains constant, we estimate that by 2025 there will be an additional 353 000 older people with substantial dependencies; they will have complex care needs that require sustained input from family carers or community health or social care teams to support independent living.^8^ In countries where publicly funded social care is means tested, such as England, older people with substantial savings or incomes will be required to pay for their care privately or rely on intensive unpaid care from their family. This level of intensity of care provision affects negatively on the carer's health, but also, as the carer is often a child, their ability to remain in the workforce.

So what are the implications for health-care services of the increases in high and low dependency states in older people for the health-care services? The workforce must be competent and adequately skilled to care for an ageing population.^31^ For care home residents with complex needs, a new specialty—a nursing home physician—has been developed in the Netherlands;^32^ however, high-quality evidence is unavailable to facilitate wider implementation of such new innovative service models. For older people with low dependency living at home, family physicians and their community care teams are the first point of contact for care, and they are increasingly undertaking the majority of chronic illness management.^31^ In the UK, undergraduate training for doctors is inadequate to meet current and future population needs, and recommendations to extend core training remain as recommendations.^33^

It is at the point of low dependency, or even earlier when individuals are first encountering activity limitation, when there is the most chance of slowing down decline. Solutions might include building of intrinsic capacity through structured exercise or rehabilitation, or by the provision of appropriate aids and adaptations to compensate for loss of capacity, and delaying the onset of disabling diseases and conditions.^29^ Additional specialist services can therefore be anticipated to support the primary care workforce and provide access to rehabilitation.^31^ Evidence confirms that community-based comprehensive geriatric assessment enables more older people to remain at home for longer than they would without comprehensive geriatric assessment, yet such services are not widely implemented,^34^ and community-based clinicians need tools that are evidence based and practical to help earlier identification of those who would benefit most from such interventions.^31^

Because CFAS I and CFAS II are large population-based studies with similar study designs that include individuals who live at home, in sheltered accommodation, and in all residential care settings, our current study provides strong cohort comparisons. However, we noted four limitations. Firstly activities defining dependency were limited to those collected and were mostly assessed through self-report or proxy report, although many were interviewer observed. Secondly, response to interview was higher in CFAS I than in CFAS II, although we used inverse probability weighting to adjust for non-response biases, and levels of dependency have increased. Thirdly, life expectancies were based on the cross-sectional population not actual observed survival data, although these findings will be available in time. Finally, there is little ethnic diversity in CFAS I and CFAS II and therefore our results have limited generalisability to non-white populations.

A 65-year-old in 2011, compared with their counterpart in 1991, spent on average almost a year longer requiring 24-h care, and a larger proportion received this care at home provided by family and friends and formal home-based services. Early intervention with rehabilitation and provision of appropriate assistive technology when low dependency is first encountered might ensure that fewer remaining years are spent with higher dependency, reducing the burden on family and state funding. Additionally, interval need is more transparent than the usual disability measures, and it fits well within the WHO framework for healthy ageing.^29^ Consensus on the items to form the measure in a global context will be important to provide a policy-relevant outcome for public health interventions and one that can be used by individuals and countries planning future care costs.

## References

[1] De Brauwer I, D'Hoore W, Swine C, Thys F, Beguin C, Cornette P (2014). Changes in the clinical features of older patients admitted from the emergency department. Arch Gerontol Geriatr.

[2] Marengoni A, Angleman S, Melis R (2011). Aging with multimorbidity: a systematic review of the literature. Ageing Res Rev.

[3] Christensen K, McGue M, Petersen I, Jeune B, Vaupel JW (2008). Exceptional longevity does not result in excessive levels of disability. Proc Natl Acad Sci USA.

[4] UN Department of Economic and Social Affairs (2009). World population ageing 2009.

[5] Appleby J, Thompson J, Jabbal J (2015). Quarterly monitoring report— how is the NHS performing?.

[6] Mathers CD, Stevens GA, Boerma T, White RA, Tobias MI (2015). Causes of international increases in older age life expectancy. Lancet.

[7] Collerton J, Davies K, Jagger C (2009). Health and disease in 85 year olds: baseline findings from the Newcastle 85+ cohort study. BMJ.

[8] Jagger C, Collerton JC, Davies K (2011). Capability and dependency in the Newcastle 85+ cohort study. Projections of future care needs. BMC Geriatr.

[9] Langa KM, Larson EB, Crimmins EM (2016). A comparison of the prevalence of dementia in the United States in 2000 and 2012. JAMA Intern Med.

[10] Matthews FE, Arthur A, Barnes LE (2013). A two-decade comparison of prevalence of dementia in individuals aged 65 years and older from three geographical areas of England: results of the Cognitive Function and Ageing Study I and II. Lancet.

[11] Jagger C, Matthews FE, Wohland P (2016). A comparison of health expectancies over two decades in England: results of the Cognitive Function and Ageing Study I and II. Lancet.

[12] Freedman VA, Wolf DA, Spillman BC (2016). Disability-free life expectancy over 30 years: a growing female disadvantage in the US population. Am J Public Health.

[13] Malley J, Hancock R, Murphy M (2011). The effect of lengthening life expectancy on future pension and long-term care expenditure in England, 2007 to 2032. Health Stat Q.

[14] Kingston A, Collerton J, Davies K, Bond J, Robinson L, Jagger C (2012). Losing the ability in activities of daily living in the oldest old: a hierarchic disability scale from the Newcastle 85+ study. PLoS One.

[15] Stineman MG, Streim JE, Pan Q, Kurichi JE, Rose SMS-F, Xie D (2014). Activity limitation stages empirically derived for activities of daily living (ADL) and instrumental ADL in the US adult community-dwelling medicare population. PMR.

[16] Isaacs B, Neville Y (1976). The needs of old people. The ‘interval’ as a method of measurement. Br J Prev Soc Med.

[17] Brayne C, Johnson T, Bond J (1999). Profile of disability in elderly people: estimates from a longitudinal population study. BMJ.

[18] Nadash P, Doty P, von Schwanenflugel M (2017). The German long-term care insurance program: evolution and recent developments. Gerontologist.

[19] Mortimer J, Green M (2015). The health and care of older people in England 2015.

[20] Folstein M, Folstein S, McHugh PR (1975). A practical method for grading the cognitive state of patients for the clinician. J Psychiat Res.

[21] McGee MA, Johnson AL, Kay DW (1998). The description of activities of daily living in five centres in England and Wales. Medical Research Council Cognitive Function and Ageing Study. Age Ageing.

[22] Sullivan DF (1971). A single index of mortality and morbidity. HSMHA Health Rep.

[23] Jagger C (1999). Health expectancy calculation by the Sullivan method: a practical guide.

[24] Jagger C, Robine JM, Rogers RG, Crimmins EM (2011). Healthy life expectancy. International handbook of adult mortality.

[25] Hashimoto S, Kawado M, Seko R (2010). Trends in disability-free life expectancy in Japan, 1995–2004. J Epidemiol.

[26] Kreft D, Doblhammer G (2016). Expansion or compression of long-term care in Germany between 2001 and 2009? A small-area decomposition study based on administrative health data. Popul Health Metr.

[27] Seko R, Hashimoto S, Kawado M (2012). Trends in life expectancy with care needs based on long-term care insurance data in Japan. J Epidemiol.

[28] Fernandez J-L, Forder J (2010). BUPA Health Pulse 2010. Ageing societies: challenges and opportunities.

[29] Beard JR, Officer A, de Carvalho IA (2016). The World report on ageing and health: a policy framework for healthy ageing. Lancet.

[30] Matthews FE, Bennett H, Wittenberg R (2016). Who lives where and does it matter? Changes in the health profiles of older people living in long term care and the community over two decades in a high income country. PLoS One.

[31] Robinson L (2015). Present and future configuration of health and social care services to enhance robustness in older age.

[32] Koopmans R, Lavrijsen JCM, Hoek F (2013). Concrete steps toward academic medicine in long term care. J Am Med Dir Assoc.

[33] Gerada C, Riley B, Simon C (2012). Preparing the future GP: the case for enhanced GP training.

[34] Ellis G, Whitehead MA, Robinson D, O'Neill D, Langhorne P (2011). Comprehensive geriatric assessment for older adults admitted to hospital: meta-analysis of randomised controlled trials. BMJ.

