# Department of Error

**DOI:** 10.1016/S0140-6736(17)32864-7

**Published:** 2018-01-06

*Kingston A, Wohland P, Wittenberg R, et al. Is late-life dependency increasing or not? A comparison of the Cognitive Function and Ageing Studies (CFAS).* Lancet *2017; **390**: 1676–84*—In this Article (published online first on Aug 15, 2017), in the last sentence of the Methods paragraph within the Summary, the population projections should be for the UK and not for England, and should read as “…the proportions in each dependency state (by age group and sex) were applied to the 2014 UK population projections”. In the Introduction section, the population projections should also be for the UK and not for England, and should read “…to model the effect of these findings on future demands for formal social care for older people in the UK”. Finally, the second to last paragraph of the Statistical analysis section should read “To project future demand for care to 2035, the proportions in each dependency state were applied to the 2014 UK population projections…” These corrections have been made to the online version as of Nov 9, 2017.

