# Supplementary material for: Is late-life dependency increasing or not? A comparison of the Cognitive Function and Ageing Studies (CFAS)
Source: Lancet. 2017 Oct 7;390(10103):1676–84. doi: 10.1016/S0140-6736(17)31575-1 (PMC5640505; doi:10.1016/S0140-6736(17)31575-1)
Supplement: Supplementary appendix [file mmc1.pdf]

THE LANCET

Supplementary appendix

This appendix formed part of the original submission and has been peer reviewed. We post it as supplied by the authors.

Supplement to: Kingston A, Wohland P, Wittenberg R, et al, on behalf of the Cognitive Function and Ageing Studies collaboration. Is late-life dependency increasing or not? A comparison of the Cognitive Function and Ageing Studies (CFAS). *Lancet* 2017; published online Aug 15. http://dx.doi.org/10.1016/S0140-6736(17)31575-1.

Supplementary material

This material formed part of the original submission and has been peer reviewed.

Supplement to: Kingston A, Wohland P, Wittenberg R, et al, on behalf of the Medical Research Council Cognitive Function and Ageing Collaboration. Is late life dependency increasing or not? A comparison of the Cognitive Function and Ageing Studies.

Supplementary Methods

Sampling

Fieldwork for CFAS I was conducted in six geographical areas of the United Kingdom (Cambridgeshire, Gwynedd, Liverpool, Newcastle, Nottingham and Oxford) between 1989 and 1994. Liverpool had a separate design from the other centres, and Gwynedd and Oxford were not included in CFAS II due to financial considerations for the new study. Three centres are included in CFAS II: Cambridgeshire (consisting of the rural area of East Cambridgeshire and Fenland centred on Ely and surrounding villages), Newcastle upon Tyne and Nottingham. The sampling frame was the same as the CFAS I enumeration; all persons aged 65 years or older registered with a GP, including individuals in institutions with the sampling strategy undertaken twice to ensure the population base was as up to date as possible. Individuals from each centre were drawn from general practice records covering the same geographical base (CFAS has kept up with changes in practices). A stratified sample based on the age groups 65-74 and 75 years and over was used with 50% of the sample in each age group and over-sampling to cope with individuals with incorrect registration and ineligibility, GP refusals, and refusals by the individuals or their carers. The geographical sequencing in CFAS I was replicated in CFAS II. Any individuals previously included in the CFAS I study were available for the new cohort study as this is a complete re-enumeration of the population and the last follow up of the first cohort was completed in 2004. The primary care practices screened records of patients in selected samples regularly for deaths and terminal illness.

Approach

Individuals who had been ascertained as eligible from the general practitioners database received, after approval from the GP, an introductory letter, patient information sheet and photograph of the interviewer who would visit them at their current residence (own home or care home) within seven days of the receipt of the study information. The aim of the initial approach by the interviewer was to discuss the study and provide more detail where necessary to enable them to make an informed decision regarding participation. If they wished to take part an appointment was made to return at a convenient time at which written informed consent was obtained immediately prior to the interview. Where cognitive impairment was judged to limit the ability of participants to provide reliable answers, proxy

informants, identified by the participant, were used. Proxies primarily consisted of spouses, offspring or occasionally paid-for carers. Interviewers were recruited and trained, initially at Cambridge with completion at each geographical centre using the methods developed for CFAS I.

All research interviewers underwent the necessary checks with the structures of that time (enhanced Criminal Records Bureau clearance, Research Passports/Letters of access from their local Primary Care Trusts). The comprehensive training covered the Data Protection Act 1998 and the Mental Capacity Act 2005. Following training, interviewers undertook a number of practice interviews with volunteers, recordings of these interviews were reviewed by the Senior Study Coordinator with detailed feedback before independent interviewing began. Checks of all paperwork and data with intermittent taped interviews with feedback to the interviewers continued throughout the fieldwork. All centres undertook slightly more than the planned 2,500 individuals due to the process of contact already having started when the centre reached 2,500 completed interviews.

Sample size

The target sample of 2,500 at each site allowed estimation of overall sex-specific prevalence rate of dementia to within 2% in each centre, and is sufficient to test equivalence of proportions between centres to within a margin of 3%. The sites chosen represent the range found within CFAS I with the healthiest patterns of ageing in Cambridgeshire, the worst in Newcastle upon Tyne and Nottingham lying between. Thus it will be possible to see whether these patterns are replicated over time.

Supplementary Results

Supplementary Table 1: Dependency by age, gender, residential status and cohort

		All		Community		Care home	
		**CFAS I**	**CFAS II**	**CFAS 1**	**CFAS II**	**CFAS 1**	**CFAS II**
		%	%	%	%	%	%
**65-74**							
**MEN**							
	**Independent**	82·9	76·1	83·5	76·3	20·0	29·0
	**Low dependency**	12·2	15·9	12·1	15·9	20·2	18·3
	**Medium dependency**	3·0	3·6	2·8	3·6	26·3	14·5
	**High dependency**	1·9	4·3	1·6	4·2	33·5	38·2
**WOMEN**							
	**Independent**	73·0	69·2	73·6	69·4	12·6	0·0
	**Low dependency**	23·1	24·5	23·2	24·5	0·0	0·0
	**Medium dependency**	2·4	3·4	2·2	3·3	25·2	42·8
	**High dependency**	1·5	2·9	1·0	2·8	62·3	57·2
**75-84**							
**MEN**	**Independent**	62·3	60·7	63·4	61·6	13·5	5·2
	**Low dependency**	25·4	25·9	25·4	26·3	26·1	5·8
	**Medium dependency**	8·7	7·2	8·6	7·0	13·3	21·2
	**High dependency**	3·6	6·2	2·7	5·1	47·1	67·8
**WOMEN**	**Independent**	46·1	39·7	48·1	40·5	5·9	3·1
	**Low dependency**	41·4	49·3	43·0	50·3	8·1	3·9
	**Medium dependency**	7·9	5·7	6·7	5·4	30·7	18·3
	**High dependency**	4·7	5·3	2·2	3·8	55·4	74·6
**85+**							
**MEN**	**Independent**	29·1	28·8	32·5	30·1	0·0	0·0
	**Low dependency**	41·2	45·6	45·5	46·6	12·1	22·9
	**Medium dependency**	19·9	16·4	17·8	16·4	33·9	17·9
	**High dependency**	9·8	9·2	3·2	6·9	54·0	59·2
**WOMEN**	**Independent**	14·1	11·8	17·0	13·5	1·7	1·2
	**Low dependency**	53·1	57·6	63·5	65·2	8·9	11·1
	**Medium dependency**	19·7	14·7	15·3	13·7	39·1	20·5
	**High dependency**	13·1	15·9	4·2	7·6	51·3	67·2
**ALL**	**Independent**	60·5	55·4	62·8	56·7	5·3	3·0
	**Low dependency**	28·7	32·4	29·5	32·9	10·4	10·2
	**Medium dependency**	6·8	6·3	5·8	6·0	32·6	20·3
	**High dependency**	3·9	5·9	2·0	4·4	51·7	66·5

Supplementary Table 2: Life expectancy and years spent in different care states at age 85 in 1991 and 2011 and change between 1991 and 2011, by sex (95%CI in parentheses)

	1991	2011	Difference 2011-1991
MEN aged 85			
Life Expectancy (LE)	4·0	5·6	1·6
Years independent (ILE)	1·2 (0·7-1·6)	1·4 (0·9-1·9)	0·3 (-0·4-1·0)
Years with low dependency (LDLE)	1·6 (1·1-2·1)	2·4 (1·9-2·8)	0·7 (0·1-1·4)
Years with medium dependency (MDLE)	0·8 (0·3-1·3)	0·8 (0·3-1·4)	0·0 (-0·7-0·8)
Years with high dependency (HDLE)	0·4 (-0·1-0·9)	0·9 (0·4-1·5)	0·5 (-0·2-1·3)
Proportion (%) of LE spent			
Independent	28·8 (16·7-41·0)	25·6 (16·2-34·9)	-3·3 (-18·6-12·1)
With low dependency	40·8 (29·8-51·9)	42·6 (34·3-50·9)	1·7 (-12·0-15·5)
With medium dependency	20·5 (7·8-33·2)	15·1 (5·3-24·8)	-5·4 (-21·5-10·6)
With high dependency	10·0 (-3·7-23·7)	16·7 (7·0-26·5)	6·7 (-10·1-23·5)
WOMEN aged 85			
Life Expectancy (LE)	4·6	7·1	2·5
Years independent (ILE)	0·7 (0·3-1·1)	0·7 (0·2-1·2)	0·0 (-0·6-0·7)
Years with low dependency (LDLE)	2·5 (2·2-2·8)	3·7 (3·3-4·1)	1·2 (0·7-1·6)
Years with medium dependency (MDLE)	0·9 (0·5-1·2)	0·9 (0·4-1·4)	0·0 (-0·6-0·6)
Years with high dependency (HDLE)	0·6 (0·2-0·9)	1·8 (1·4-2·3)	1·3 (0·7-1·9)
Proportion (%) of LE spent			
Independent	14·8 (7·4-22·2)	10·2 (2·8-17·5)	-4·6 (-15·1-5·8)
With low dependency	53·9 (48·5-59·4)	51·6 (46·1-57·1)	-2·3 (-10·1-5·5)
With medium dependency	19·0 (11·7-26·4)	12·5 (5·1-19·9)	-6·6 (-17·3-3·9)
With high dependency	12·5 (4·9-20·1)	25·9 (19·0-32·8)	13·4 (3·1-23·6)
